# Glycemic Variability Assessed by Continuous Glucose Monitoring and Short-Term Outcome in Diabetic Patients Undergoing Percutaneous Coronary Intervention: An Observational Pilot Study

**DOI:** 10.1155/2015/250201

**Published:** 2015-07-26

**Authors:** Annunziata Nusca, Angelo Lauria Pantano, Rosetta Melfi, Claudio Proscia, Ernesto Maddaloni, Rocco Contuzzi, Fabio Mangiacapra, Andrea Palermo, Silvia Manfrini, Paolo Pozzilli, Germano Di Sciascio

**Affiliations:** ^1^Department of Cardiovascular Sciences, Campus Bio-Medico University of Rome, Via Alvaro del Portillo 200, 00128 Rome, Italy; ^2^Department of Endocrinology and Diabetes, Campus Bio-Medico University of Rome, Via Alvaro del Portillo 200, 00128 Rome, Italy

## Abstract

Poor glycemic control is associated with unfavorable outcome in patients undergoing percutaneous coronary intervention (PCI), irrespective of diabetes mellitus. However a complete assessment of glycemic status may not be fully described by glycated hemoglobin or fasting blood glucose levels, whereas daily glycemic fluctuations may influence cardiovascular risk and have even more deleterious effects than sustained hyperglycemia. Thus, this paper investigated the effectiveness of a continuous glucose monitoring (CGM), registering the mean level of glycemic values but also the extent of glucose excursions during coronary revascularization, in detecting periprocedural outcome such as renal or myocardial damage, assessed by serum creatinine, neutrophil gelatinase-associated lipocalin (NGAL), and troponin I levels. High glycemic variability (GV) has been associated with worse postprocedural creatinine and NGAL variations. Moreover, GV, and predominantly hypoglycemic variations, has been observed to increase in patients with periprocedural myocardial infarction. Thus, our study investigated the usefulness of CGM in the setting of PCI where an optimal glycemic control should be achieved in order to prevent complications and improve outcome.

## 1. Introduction

Diabetes mellitus represents a major risk factor for the development of coronary artery disease [1, 2] and an important predictor of outcome in patients undergoing percutaneous coronary intervention (PCI) [3, 4].

Recent studies have suggested a potential and independent prognostic role of preprocedural blood glucose levels (BGLs), irrespective of diabetes mellitus, in patients undergoing PCI; both hyperglycemia and hypoglycemia significantly correlated with the incidence of periprocedural myocardial infarction (PMI) [5], contrast-induced acute kidney injury (CI-AKI) [5–8], and in-stent restenosis [5, 9, 10]. Potential pathophysiologic mechanisms include endothelial dysfunction and oxidative stress caused by abnormal BGLs that may increase both myocardial damage and renal toxicity of contrast media during PCI [11, 12].

However, a complete assessment of glycemic status in patients undergoing PCI may not be fully evaluated by fasting BGLs or glycated hemoglobin (HbA1c), currently considered the most prominent biomarker to evaluate glycemic control [13]; several studies showed that daily fluctuations of BGLs may influence cardiovascular risk and have even more deleterious effects than sustained hyperglycemia [14–17]. Thus, more information regarding patients glycemic control may be obtained by a continuous glucose monitoring (CGM), registering not only the mean level of glycemic values but also the extent of glucose excursions during the period in which coronary revascularization is performed. Glycemic variability assessed by CGM has been associated with the presence and severity of coronary atherosclerosis in diabetic patients [16, 17] and with endothelial dysfunction also in nondiabetic patients [18].

In the present study, we investigated for the first time the prognostic role of glycemic variability assessed by using a CGM on short-term outcome in patients with type 2 diabetes mellitus undergoing PCI, on insulin or hypoglycemic oral agents or diet treatment. In particular, we correlated the glycemic variability indexes with myocardial and renal damage markers after coronary stenting.

## 2. Methods

### 2.1. Study Population

We prospectively enrolled 28 consecutive patients with type 2 diabetes mellitus undergoing PCI at our institution between July 2012 and January 2013. Exclusion criteria were as follows: primary intervention for acute myocardial infarction; acute coronary syndrome in the previous 72 hours; left ventricular ejection fraction <30%; severe renal failure (glomerular filtration rate (GFR) < 30 mL/min/1.73 m^2^); coexistent immunological, inflammatory, or neoplastic disease at the time of enrolment; and contraindications to antithrombotic or antiplatelet therapy. Presence of diabetes mellitus was defined as history of diabetes controlled by diet or oral hypoglycemic agents or insulin. Coronary intervention was performed with standard technique. Aspirin 100–325 mg and clopidogrel 600 mg were given at least 12 hours before the procedure. Patients with chronic renal failure (GFR < 60 mL/min/1.73 m^2^) underwent intravenous periprocedural hydration with normal saline (1 mL/hour/kg body weight for at least 12 hours before and 24 hours after intervention). Contrast agent used in all procedure was iodinated, nonionic, low-osmolality contrast medium, iobitridol. The procedure was considered successful if there was <30% residual stenosis in the target lesion, with TIMI (Thrombolysis in Myocardial Infarction) grade III flow and in the absence of major in-hospital complications: death, myocardial infarction, or urgent coronary revascularization (re-PCI or coronary artery bypass graft). All subjects enrolled in this study provided written informed consent. The study was approved by our local ethical committee.

### 2.2. Continuous Glucose Monitoring and Glycemic Variability Indexes

**Figure 1:**
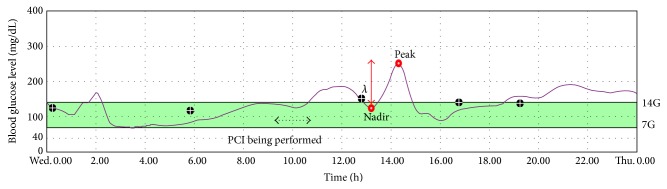
Example of 24-hour continuous glucose monitoring with* i*Pro.

All patients were equipped with* i*Pro continuous glucose recorder (Medtronic, Northridge, CA) and monitored for 48 consecutive hours after admission. A CGM sensor (Enlite Sensor) was inserted into the subcutaneous abdominal fat tissue and calibrated according to the standard Medtronic operating guidelines. The* i*Pro continuous glucose recorder measures subcutaneous tissue interstitial glucose levels continuously, recording values every 5 minutes, within a range 40–400 mg/dL. During* i*Pro CGM, patients checked their blood glucose level with a self-monitoring of blood glucose at least 4 times per day. The FreeStyle Lite (Abbott Laboratories, Abbott Park, IL) BG-monitoring system was used to calibrate the* i*Pro continuous glucose recorder. After monitoring for 48 hours, the recorded data were downloaded for analysis of the glucose profile and glucose excursion parameters with CareLink* i*Pro System. Analysis was performed on data obtained in the period between 12 hours before and 12 hours after PCI. Intraday GV was expressed by the glycemic variability indexes reported in Table 1 and Figure 1 [19, 20].

### 2.3. Laboratory Assays

Serum creatinine (SCr) was measured at hospital admission, 6 and 24 hours after PCI and thereafter if clinically indicated. The estimated GFR was calculated by the Modification of Diet in Renal Disease Study (MDRD) equation. CI-AKI was defined as an absolute increase in SCr ≥ 0.3 mg/dL within 24 hours after contrast exposure [21].

In 25 patients blood samples were also collected before and 6 hours after the procedure for the determination of the neutrophil gelatinase-associated lipocalin (NGAL) by using the NGAL Rapid ELISA kit (BioPorto Diagnostics).

Creatine kinase-MB (CK-MB) and troponin I (TnI) levels were measured at the time of intervention, 6 and 24 hours after PCI, and thereafter if clinically indicated, according to standard enzymatic procedures (LOCI immunochemiluminometric assay, SIEMENS). The laboratory upper limits of normal (ULN, the 99th percentile of normal population with a total imprecision of 10%) were 3.6 ng/mL for CK-MB and 0.05 ng/mL for TnI. PMI was defined by elevation of TnI (>5 × 99th percentile Upper Reference Limit (URL)) in patients with normal baseline values (≤99th percentile URL) or an increase of TnI > 20% if the baseline values were elevated, in addition to either symptoms suggestive of myocardial ischaemia or new ischaemic ECG changes or angiographic findings consistent with a procedural complication or imaging demonstration of new loss of viable myocardium or new regional wall motion abnormality, according to the recently accepted third universal definition of myocardial infarction [22].

Moreover, in all patients fasting BGLs and HbA1c were obtained.

### 2.4. Statistical Analysis

Data are presented as frequencies and percentages for categorical variables and mean ± SD or median and first and third quartiles, when appropriate, for continuous variables. The Kolmogorov-Smirnov test was used to identify potential deviations from the normal distribution. Correlation between normally distributed continuous variables was determined by Pearson correlation coefficients, whereas Spearman correlation coefficients were used to analyze not normally distributed variables. The Student *t*-test and the nonparametric Mann-Whitney test were used to investigate differences between values for normally and not normally distributed variables. For parametric variables, univariate analysis was done by using linear regression analysis. Multiple regression analysis (stepwise forward selection), including variables with *p* < 0.10 at the linear regression analysis, was then performed to assess the strength and independency of associations between variables. Binary regression analysis was performed to identify the relative risk of glycemic variability on PMI occurrence. A value of *p* < 0.05 was considered statistically significant. Statistical analysis was performed using the SPSS 16.0 package for Windows.

## 3. Results

Baseline clinical features of the study population are reported in Table 2. Tables 3 and 4 show angiographic/procedural characteristics and mean values of GV indexes, respectively.

### 3.1. Glycemic Variability and Postprocedural Renal Impairment

**Figure 2:**
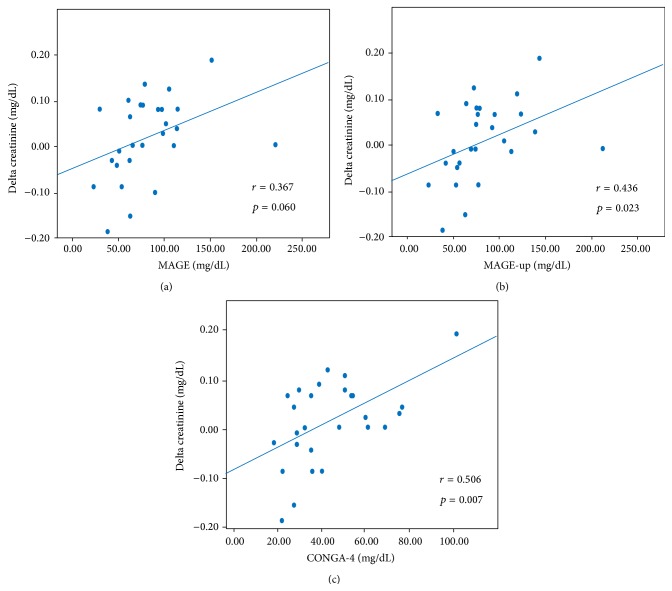
Correlation between periprocedural glycemic variability (expressed as MAGE, MAGE-up, and CONGA-4) and postprocedural serum creatinine variation. CONGA-4: continuous overall net glycemic action at 4 hours; MAGE: mean amplitude glycemic excursions; MAGE-up: mean amplitude glycemic nadir-to-peak excursions.

Analyzing glycemic variables derived from* i*Pro GCM and renal function parameters, SCr variation (ΔSCr = postprocedural SCr peak − preprocedural SCr) significantly correlated with SD (*r* = 0.440, *p* = 0.022), MAGE-up (*r* = 0.436, *p* = 0.023), and CONGA-4 (*r* = 0.506, *p* = 0.007); a positive association was also observed with MAGE (*r* = 0.367, *p* = 0.060) (Figure 2).

At the multivariate analysis, including SD, MAGE, MAGE-up, CONGA-4, and amount of contrast media (*p* < 0.10 at the univariate regression analysis), the only independent predictor of renal function deterioration was CONGA-4 (*p* = 0.030). In an additional multivariate model, in which age, baseline creatinine, and left ventricle ejection fraction were added into the model, CONGA-4 remained an independent predictive value for ΔSCr (*p* = 0.036).

**Figure 3:**
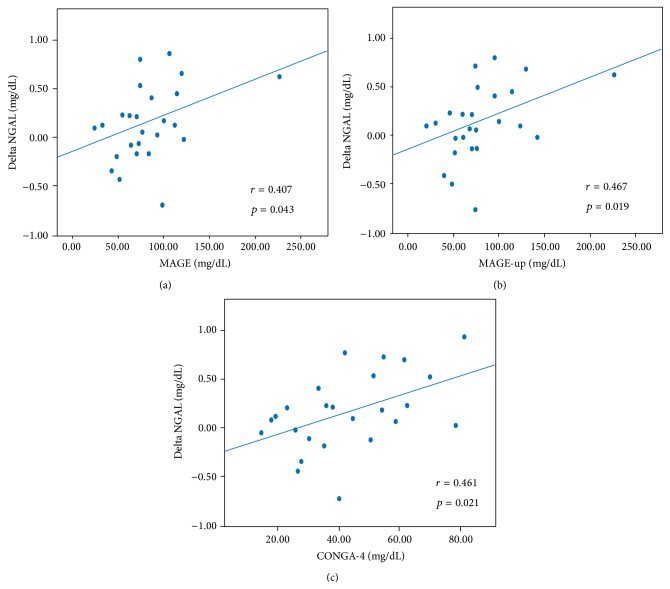
Correlation between periprocedural glycemic variability (expressed as MAGE, MAGE-up, and CONGA-4) and postprocedural NGAL variation. CONGA-4: continuous overall net glycemic action at 4 hours; MAGE: mean amplitude glycemic excursions; MAGE-up: mean amplitude glycemic nadir-to-peak excursions.

Similar results were observed for NGAL levels; a positive association was found between ΔNGAL (6 h NGAL – preprocedural NGAL) and CV (*r* = 0.404, *p* = 0.045), SD (*r* = 0.408, *p* = 0.043), MAGE (*r* = 0.407, *p* = 0.043), MAGE-up (*r* = 0.467, *p* = 0.019), and CONGA-4 (*r* = 0.461, *p* = 0.021) (Figure 3). CONGA-4 maintained a predictive value at the multivariate analysis performed including age, contrast amount, left ventricle ejection fraction, and baseline creatinine (*p* = 0.042).

A significant correlation was observed between ΔSCr and ΔNGAL (*r* = 0.417, *p* = 0.043), whereas HbA1c levels were not significantly associated with postprocedural SCr or NGAL variations. In the overall population no patient developed CI-AKI.

### 3.2. Glycemic Variability and Postprocedural Myocardial Damage

**Figure 4:**
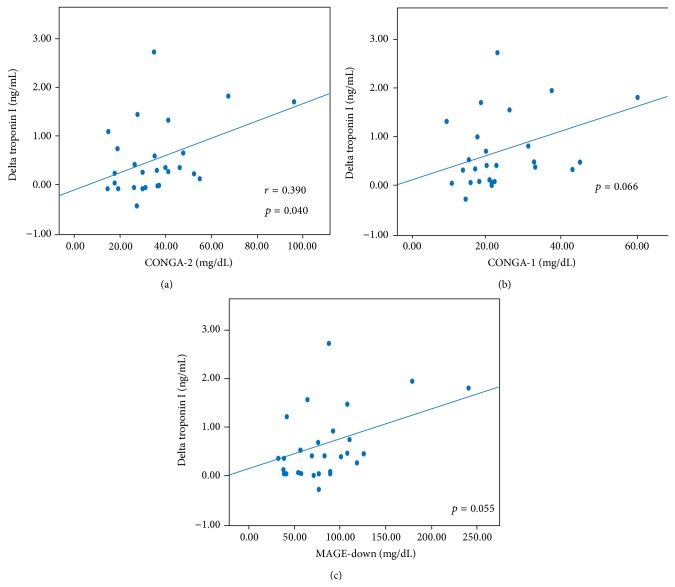
Correlation between periprocedural glycemic variability (expressed as CONGA-2, CONGA-1, and MAGE-down) and postprocedural troponin I increase. CONGA-1: continuous overall net glycemic action at 1 hour; CONGA-2: continuous overall net glycemic action at 2 hours; MAGE-down: mean amplitude glycemic peak-to-nadir excursions.

**Figure 5:**
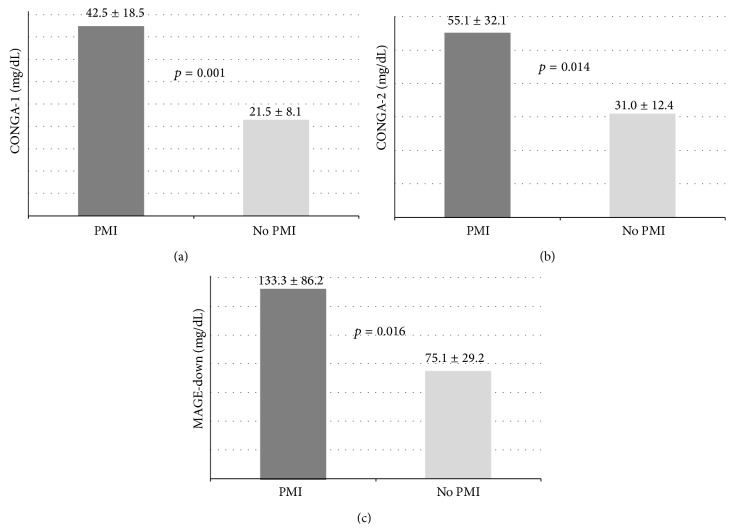
Glycemic variability indexes in patients with and without PMI. CONGA-1, CONGA-2, and MAGE-down values in patients with and without PMI. CONGA-1: continuous overall net glycemic action at 1 hour; CONGA-2: continuous overall net glycemic action at 2 hours; MAGE-down: mean amplitude glycemic peak-to-nadir excursions; PMI: periprocedural myocardial infarction.

Postprocedural TnI increase (ΔTnI = postprocedural TnI peak − preprocedural TnI) significantly correlated with CONGA-2 (*r* = 0.390, *p* = 0.040); however a trend was observed also for CONGA-1 (*p* = 0.066) and MAGE-down (*p* = 0.055) (Figure 4). Notably, HbA1c levels did not correlate with postprocedural TnI variations. The incidence of PMI in the study population was 11% (3 patients). These patients showed significantly higher mean values of CONGA-1, CONGA-2, and MAGE-down, during CGM, compared to patients not suffering from this complication (Figure 5).

Binary logistic regression analysis revealed that CONGA-1 was an independent risk factor for PMI occurrence (*p* = 0.041).

## 4. Discussion

This prospective study evaluates the prognostic usefulness of CGM in patients undergoing PCI. We observed a significant correlation between GV indexes assessed by CGM and renal function deterioration after contrast exposure detected by postprocedural SCr and NGAL variations. Moreover, high GV was also associated with periprocedural myocardial damage expressed by troponin release.

### 4.1. A Novel Approach to Glycemic Status Assessment

Optimal glycemic control may represent another important challenge in patients undergoing PCI, irrespective of diabetes; however, debate remains about which parameter is the most appropriate and prognostically useful to assess overall patient glycemic status. In diabetic patients, HbA1c is currently the most used biomarker to evaluate glycemic control and to guide appropriate therapeutic changes [23]. Nevertheless, HbA1c, other than limitations relating to a variety of physiological and pathological conditions that may influence its concentration, is an inappropriate marker for detecting rapid glucose changes such as acute hyperglycemia and hypoglycemia [13]. Otherwise, there is increasing evidence that especially these acute glycemic abnormalities may contribute to unfavourable outcome in patients with coronary artery disease [16, 17, 24]. Acute hyperglycemia, even in absence of diabetes, was a significant and independent predictor of CI-AKI in patients undergoing primary PCI [6, 7]. Furthermore, a significant association between preprocedural BGLs (hyperglycemia and hypoglycemia) and PMI was observed in patients undergoing elective PCI [5]. However, these previous studies successfully investigated the prognostic role of a spot BGLs detection on admission to intensive care or before coronary revascularization; however, a single glucose value cannot estimate overall GV, that is, all acute fluctuations of glucose levels from peaks to nadirs and vice versa during in-hospital stay. This concept should be carefully considered in particular conditions, such as critical illness and coronary revascularization, where other factors (stress, pain/discomfort, and nutrition discontinuation) may further increase acute glucose variations. Thus, CGM registering BGLs continuously may provide more detailed information regarding GV.

GV assessed by CGM has been demonstrated to correlate significantly with endothelial dysfunction, measured by brachial artery flow-mediated dilation and carotid intima-media thickness in diabetic and nondiabetic patients [18, 25]. Moreover, GV, expressed as MAGE, exhibited a more specific triggering effect on oxidative stress, estimated from 24-hour urinary excretion rates of free 8-iso prostaglandin F_2_ *α*, than chronic sustained hyperglycemia [26]. A recent study observed that MAGE was an independent predictor of coronary disease in patients with hyperglycemia, even more than HbA1c [16, 17]. These findings confirmed the significant prognostic impact of GV on cardiovascular outcome in diabetic patients, despite normal BGLs and HbA1c values, suggesting a possible deleterious effect also on patients without diabetes mellitus. Of note, in our study, HbA1c levels did not correlate with troponin or renal damage markers.

### 4.2. Glycemic Variability and Its Impact on Myocardial and Renal Damage

Mechanisms by which glucose abnormalities may be a causal factor for poor outcome in patients undergoing PCI remain not completely understood. Hyperglycemia causes release of proinflammatory cytokines (IL-6, IL-8, IL-18, and TNF-*α*), diminished bioavailability of nitric oxide with attendant endothelial dysfunction, and increased production of oxygen-derived free radicals with enhanced oxidative stress [11, 12, 27]. All these mechanisms have also been described in the pathogenesis of renal damage after contrast media exposure [28]; thus, acute hyperglycemia may exacerbate the deleterious effects of contrast agents on the kidney. Notably, in our study, we observed a significant correlation between MAGE-up, predominantly indicating hyperglycemic “peaks,” and both postprocedural SCr and NGAL variations. Conversely, hypoglycemia and rapid changes in BGLs have been shown to increase epinephrine and norepinephrine levels, which may induce vasoconstriction, platelet activation, enhanced vascular inflammation, and endothelial dysfunction [29]; all these factors may worsen periprocedural myocardial damage in the setting of PCI [5]. These mechanisms may partially explain the correlation observed in our study between MAGE-down, indicating hypoglycemic nadirs, and CONGA-2, detecting small glycemic swings occurring over a short-time interval, and troponin release after coronary stenting. Based on these data, we may hypothesize that myocardial injury could be primarily influenced by hypoglycemia and by rapid glycemic spikes, whereas the kidney may be mainly susceptible to slower and longer hyperglycemic excursions, as suggested by the prognostic independent role of CONGA-4 in postprocedural creatinine variations observed in our study. However, hyperglycemia and hypoglycemia are dynamic conditions and connected to each other in the overall concept of GV.

### 4.3. CGM as a Useful Tool for an Optimal Glycemic Control

Given the growing body of evidence indicating the prognostic role of glucose abnormalities, considerable attention has been focused on determining whether optimal glycemic control may lead to improved cardiovascular outcome. Several studies have questioned the safety and effectiveness of a tight glycemic control by infusion of insulin or glucose insulin potassium (GIK), especially in critically ill patients such as those with acute MI, providing conflicting results [30–32]. The reduced benefit of intensive glucose lowering strategy in the above cited studies may be partially explained by the fact that they aimed to control HbA1c levels or fasting BGLs, without continuous monitoring of GV. Moreover, a more aggressive glucose control has also been associated with an increased incidence of hypoglycemia, a proven independent risk factor for cardiovascular mortality [33, 34]. A recent subanalysis from the Normoglycaemia in Intensive Care Evaluation and Survival Using Glucose Algorithm Regulation (NICE-SUGAR) trial confirmed that, among critically ill patients, moderate and severe hypoglycemia due to intensive glycemic control are both strongly associated with an increased risk of death (OR 1.41 and 2.10, resp.) [35]. In this setting, optimal glycemic management requires judicious treatment of hyperglycemia avoiding hypoglycemia; for this purpose, CGM may help avoid excessive glycemic variability and prevent hypoglycemic episodes by providing continuous information on glycemic trends.

## Study Limitations

Some limitations of the present study have to be acknowledged. The small sample size may undoubtedly limit the value of our statistical findings; however, this is a characteristic of all pilot exploratory investigations such as ours. Obviously, we cannot prove causality between GV and renal and myocardial outcomes. The best measure of GV among all those glycemic indexes evaluated by CGM and the optimal target range remain unsolved clinical issues. Probably an appropriate range of glycemic control should be individualized according to patients' instability, diabetic status, and baseline glycemic levels. Finally, in most patients we obtained SCr levels 24 hours after contrast exposure; given that creatinine may increase up to 48 hours after contrast administration, in our study renal damage may be underestimated; however, we also evaluated NGAL at 6 hours, an earlier and more sensitive marker of renal injury [36, 37].

In conclusion, our study suggests a significant impact of glycemic variability on short-term outcome of patients undergoing coronary stenting, encouraging the use of GCM in the setting of PCI, where an optimal glycemic control should be achieved, especially in patients with diabetes mellitus.

## Figures and Tables

**Table 1:** Indexes of glycemic variability.

Measure	Formulae	Variables	Significance	Advantages	Limits
Standard deviation (SD)	$\sqrt{\frac{\sum_{i=1}^{N}{\left(x_{i}-\langle x\rangle \right)}^{2}}{N}}$	*x* = mean of glycemic observations *N* = number of observations	Variation or dispersion from the average	Easy to determine	It does not weigh greater and lower glycemic excursions; it does not address non-Gaussian skewed asymmetrical distribution or outliers

Coefficient of variability (CV)	$\frac{\text{SD}}{x}$	SD = standard deviation *x* = mean of glycemic observations	Normalized measure of dispersion from the average	Easy to determine	Same limitations as SD

Mean amplitude of glycemic excursions (MAGE)	$\sum \frac{\lambda }{x}\;\;\text{if}\;\;\lambda >v$	*λ* = blood glucose changes from peak to nadir *x* = number of valid observations *v* = 1 SD for a 24-h period	Average size of glycemic excursions (MAGE-up: from nadirs to peaks, indicating hyperglycemic fluctuations; MAGE-down: from peaks to nadirs, indicating hypoglycemic fluctuations)	It weighs hypoglycemic and hyperglycemic fluctuations equivalently	The definition of “significant” glycemic peaks and nadirs is arbitrary

Continuous overall net glycemic action (CONGA-*n*)	$\sqrt{\frac{\sum_{t=t_{1}}^{t_{k}}{\left(D_{t}-\overset{-}{D}\right)}^{2}}{k-1}}$, $\overset{-}{D}=\frac{\sum_{t=t_{1}}^{t_{k}}D_{t}}{k}$, *D*_*t*_ = *G*_*t*_ − *G*_*t*−*m*_	*t* = time *k* = number of observations *G* = number of observations over *n* × 60 min from a predetermined time	Intraday glycemic swings occurring over predetermined intervals (CONGA-1 = 1-hour interval; CONGA-2 = 2-hour interval; CONGA-4 = 4-hour interval)	Accurate measure of intraday glycemic variability	Difficult to calculate

**Table 2:** Baseline population characteristics.

Variable	*N* = 28
Female gender	3 (10)
Age (years)	67 ± 8
Waist (cm)	103 ± 9
BMI (kg/m^2^)	28 ± 4
Diabetes mellitus	
Insulin treated	3 (11)
OHD treated	20 (71)
OHD and Insulin	5 (18)
Mean duration of diabetes mellitus (years)	12 ± 6
Dyslipidemia	25 (86)
Hypertension	25 (86)
Smoking	7 (24)
Clinical presentation	
Stable angina	24 (86)
Unstable angina	4 (14)
Previous MI	7 (25)
Previous PCI	15 (53)
Previous CABG	6 (21)
LVEF (%)	56 ± 5
Medications	
Aspirin	28 (100)
Clopidogrel	28 (100)
ACEi/ARBs	14 (50)
*β*-blockers	17 (59)
Statins	21 (74)
Calcium channel blockers	10 (34)
Oral hypoglycemic agents	25 (89)
Biguanides	12 (43)
Sulphonylurea	6 (21)
Biguanides and Sulphonylurea	7 (25)

Values are given as mean ± SD or *n* (%). ACEi/ARBs: angiotensin converting enzyme inhibitors/angiotensin converting enzyme blockers; BMI: body mass index; CABG: coronary artery bypass graft; IGT: impaired glucose tolerance; LVEF: left ventricular ejection fraction; MI: myocardial infarction; OHD: oral hypoglycemic drugs; PCI: percutaneous coronary intervention.

**Table 3:** Angiographic and procedural characteristics.

Variable	*N* = 28
Multivessel disease	14 (50)
Multivessel PCI	11 (39)
Contrast media (mL)	180 ± 77
Number of treated vessels/patient	1.5 ± 0.6
Stenosis (%)	76 ± 6
B2/C type lesion^*∗*^	17 (61)
Bifurcations	9 (32)
Number of stents/patient	2.0 ± 1.4
DES implantation (%)	21 (75)
Stent diameter (mm)	2.9 ± 0.71
Stent length (mm)	17.6 ± 6.2

Values are given as mean ± SD or *n* (%). DES: drug eluting stent; PCI: percutaneous coronary intervention. ^*∗*^Ellis modification of the ACC/AHA lesion classification system [38].

**Table 4:** Glycemic indexes and laboratory assays.

Variable	*N* = 28
HbA1c (%)	7.1 ± 1.5
Fasting BGLs (mg/dL)	124 ± 48
*Preprocedural values *	
Creatinine (mg/dL)	0.86 ± 0.28
Creatinine clearance (mL/min/1.73 m^2^)	101 ± 34
NGAL (pg/mL)	1.5 ± 1.0
Troponin I (ng/mL)	0.13 ± 0.27
*CGM values *	
Max BGL (mg/dL)	254 ± 62
Min BGL (mg/dL)	63 ± 14
Total average BGL (mg/dL)	126.8 ± 34.6
Total SD BGL (mg/dL)	33 ± 15.8
Glycemic CV (%)	25.9 ± 8.7
MAGE (mg/dL)	80 ± 40
MAGE-up (mg/dL)	80 ± 40
MAGE-down (mg/dL)	60 ± 70
CONGA-1 (mg/dL)	23.7 ± 11.3
CONGA-2 (mg/dL)	33.6 ± 16.4
CONGA-4 (mg/dL)	43.3 ± 20.3

Values are given as mean ± SD or *n* (%). BGL: blood glucose level; CGM: continuous glucose monitoring; CONGA (1, 2, 4): continuous overall net glycemic action (at 1 hour, 2 hours, and 4 hours); CV: coefficient of variability; HbA1c: glycated haemoglobin; MAGE: mean amplitude glycemic excursions; NGAL: neutrophil associated gelatinase lipocalin; PCI: percutaneous coronary intervention; SD: standard deviation.

## Abbreviations

BGLs: Blood glucose levels
CGM: Continuous glucose monitoring
CI-AKI: Contrast-induced acute kidney injury
CK-MB: Creatine kinase-MB
CONGA: Continuous overall net glycemic action
CV: Coefficient of variability
GFR: Glomerular filtration rate
GIK: Glucose insulin potassium
HbA1c: Glycated hemoglobin
MAGE: Mean amplitude glycemic excursions
NGAL: Neutrophil gelatinase-associated lipocalin
PCI: Percutaneous coronary intervention
PMI: Periprocedural myocardial infarction
SCr: Serum creatinine
SD: Standard deviation
Tn I: Troponin I.
